# Introduction to the themed collection on ‘Molecular and Nanotheranostics’

**DOI:** 10.1039/d3cb90050a

**Published:** 2024-01-12

**Authors:** Thimmaiah Govindaraju

**Affiliations:** a Bioorganic Chemistry Laboratory, New Chemistry Unit, and School of Advanced Materials (SAMat), Jawaharlal Nehru Centre for Advanced Scientific Research (JNCASR) Jakkur P.O. Bengaluru 560064 Karnataka India tgraju@jncasr.ac.in

## Abstract

Thimmaiah Govindaraju introduces the themed collection on molecular and nanotheranostics.
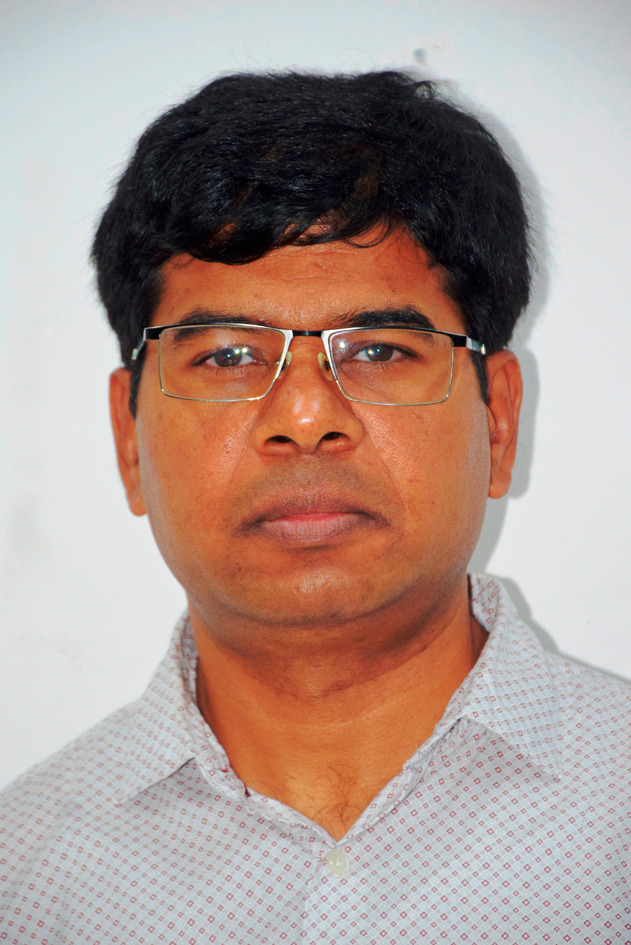

The integrated approach of theranostics has the potential to advance biomedical research towards achieving the goals of personalized medicine. Theranostics provides a holistic solution for disease management by combining diagnostic and therapeutic modalities. In this diagnostic therapy, molecular and/or material tools for treatment and diagnostic imaging are strategically embedded into a single system. These multifunctional diagnostic platforms carrying therapeutic and target tracking agents offer remedial effects and imaging of markers in tissue or organs, which are used to assess disease staging, treatment planning, and therapeutic efficacy. Radiopharmaceutical, nano, and macromolecular systems armed with tools to detect, treat, track, and image biomolecular targets in real-time have been developed as theranostics to effectively manage disease progression and cures. In recent years, the field has witnessed the emergence of small molecules and small molecular conjugates as theranostic tools.

This themed collection of articles covers advancements in molecular and nanotheranostics with particular emphasis on the design of theranostic tools and their selective interaction with biomolecular targets to image and ameliorate pathological conditions. This themed collection is anticipated to catalyze the development of precision theranostics as advanced and personalizable tools in chemical biology and modern medicine.

Alzheimer’s disease (AD) is a multifactorial neurodegenerative disease that lacks effective diagnosis and treatment. In their review, Wang and co-workers discuss the progress made in the development of small-molecule-based theranostic agents for AD (https://doi.org/10.1039/D3MD00330B). The review focuses on theranostic agents targeting amyloid beta (Aβ), which are discussed under different categories based on their diagnostic modalities: fluorescence, positron emission tomography (PET), and magnetic resonance imaging (MRI)-guided theranostic agents. The authors discuss the design rationales, chemical structures, and working mechanisms. While the opportunities for small-molecule-based theranostic agents in AD are highlighted, the authors also critically discuss limitations of the existing theranostic tools in terms of design, blood–brain barrier permeability, and clinical translation, and offer directions for future developments.

Integration of imaging in therapy can significantly improve the prognosis of devastating disease conditions such as cancer and neurodegeneration. Theranostic agents have a significant role to play in early-stage diagnosis, drug development, clinical-stage validation, disease treatment, and management. Govindaraju and colleagues discuss the pros and cons of existing and evolving theranostic approaches encompassing nuclear medicine, nano, polymer, dendrimer, antibody, and gene formulations, then comprehensively discuss the role of small molecules and their conjugates-based theranostics for cancer and AD (https://doi.org/10.1039/D3CB00073G). It is imperative to carefully evaluate and refine the sensitivity, specificity, and quantifiability of imaging to translate theranostic platforms to clinical use. The development of small molecule theranostics requires a synergy of advancements in synthetic organic chemistry, biology, and biomedical research. The discovery and validation of accessible markers for specific diseases, and reliable drug uptake/release/activation of imaging modalities would greatly enhance the efficacy of theranostic tools. To utilize the versatility of theranostics, the right balance of molecular complexity and optimal efficiency, and developing a synergy between dose response and diagnostic requirements, need utmost attention.

Kim and colleagues report a single-chain multi-color reporter imaging template for monitoring steroid hormonal activities at different optical spectra in subcellular compartments of live cells (https://doi.org/10.1039/D3CB00077J). The authors evaluated marine luciferases and luciferins for their spectral emission, signal intensity, substrate specificity, and subcellular distribution in mammalian cells. This study revealed that luciferases ALuc16 and ALuc49 are brightest when reacting with nCTZ as a substrate in live MDA-MB231 cells, while R86SG showed an extremely bright signal upon reacting with the substrate BBlue2.3 in the same type of cells. Based on this knowledge, the team developed two single-chain reporter imaging templates, iRFP-R86SG-NLS and A16-mNep-MLS, for locating subcellular molecular events in eukaryotic cells. iRFP-R86SG-NLS is directed to the nucleus by NLS, while A16-mNep-MLS is directed to the endoplasmic reticulum (ER) and cell membrane compartments by MLS. The probes generated bioluminescence (BL), fluorescence (FL), and bioluminescence resonance energy transfer (BRET) signals without interfering with other signals in the intracellular compartment. This modality expands the toolbox for optical probes detectable in standard microscopes and other optical imaging systems for tracking subcellular proteins and molecular events in cells.

Pascu and colleagues report fluorescent naphthalimide boronates as theranostic tools (https://doi.org/10.1039/D3CB00112A). The functional phenyl boronic acid (BA)-based fluorescent probes were developed by incorporating the 1,8-naphthalimide (NI) tag. The phenyl-boronate-NI probes were studied for their interaction with a naturally occurring polysaccharide, β-d-glucan, through *in vitro* cell imaging. The probe exhibits cell penetration and demonstrates environmental sensitivity as monitored by changes in fluorophore lifetime measurements. The cellular uptake of the probe was mediated by the presence of β-d-glucan and was utilized to visualize cancer cells using time-correlated single-photon counting (TCSPC) and two-photon fluorescence-lifetime imaging microscopy (FLIM) techniques. FLIM allowed the assessment of the effect of cellular surroundings on the probe using its fluorescence lifetime. The cancer cell-targeting through specific probe-biomolecular interactions and their monitoring using specialized imaging techniques are promising for cancer theranostics.

Jaiswal and colleagues have developed a nanocarrier that delivers active therapeutic cargos into the cytosol, bypassing endosome degradation (https://doi.org/10.1039/D3CB00090G). The biocompatible cationic dextrin (CD)-based nanoparticles (NPs) effectively deliver a therapeutic protein, cytochrome *C* (Cyt *C*), into cancer cells. This CD NPs nanocarrier design adopts a novel approach to enhance the endosomal release of the therapeutic protein. Co-delivering the nanocarrier with chloroquine enhances the endosomal escape of the therapeutic protein. The delivered Cyt *C* exhibits structural and functional activity, inducing apoptosis in HeLa cells. The mechanism of action study revealed that the externally administered Cyt *C* rapidly activates caspase-9 protein, which triggers pore formation in the mitochondria. This results in a decrease of the mitochondrial membrane potential and ultimately induces apoptosis. The engineered CD nanocarrier is demonstrated as a versatile and efficient platform for targeted protein delivery in cancer therapy. The reported strategy also holds promise in overcoming the challenges posed by resistant cancer cells that inhibit the release of Cyt *C*.

The articles presented in this collection provide valuable insight into the latest developments in the field of theranostics and are expected to catalyze further advancements in personalized medicine. I would like to thank all the authors for their exceptional contributions. Theranostics is a multidisciplinary field that involves the development, evaluation, and validation of molecular/material tools for holistic disease management by leveraging expertise in chemical biology, medicinal chemistry, biophysics/imaging, and biomedicine. Artificial intelligence and machine learning (AI and ML) tools are also expected to contribute significantly to future developments. I believe that this collection of articles will be useful for readers to understand and undertake further research in the field.

## Supplementary Material

